# Sex-differences in circulating biomarkers during acute myocardial infarction: An analysis from the SWEDEHEART registry

**DOI:** 10.1371/journal.pone.0249830

**Published:** 2021-04-08

**Authors:** Kai M. Eggers, Lars Lindhagen, Tomasz Baron, David Erlinge, Marcus Hjort, Tomas Jernberg, Nina Johnston, György Marko-Varga, Melinda Rezeli, Jonas Spaak, Bertil Lindahl

**Affiliations:** 1 Department of Medical Sciences, Uppsala University, Uppsala, Sweden; 2 Department of Medical Sciences and Uppsala Clinical Research Center, Uppsala University, Uppsala, Sweden; 3 Department of Cardiology, Clinical Sciences, Lund University, Skåne University Hospital, Lund, Sweden; 4 Division of Cardiovascular Medicine, Department of Clinical Sciences, Danderyd Hospital, Karolinska Institute, Stockholm, Sweden; 5 Department of Biomedical, Clinical Protein Science & Imaging, Engineering, Lund University, Lund, Sweden; Erasmus Medical Centre: Erasmus MC, NETHERLANDS

## Abstract

**Background:**

Sex-differences in the pathobiology of myocardial infarction are well established but incompletely understood. Improved knowledge on this topic may help clinicians to improve management of men and women with myocardial infarction.

**Methods:**

In this registry-based cohort study (SWEDEHEART), we analyzed 175 circulating biomarkers reflecting various pathobiological axes in 856 men and 243 women admitted to Swedish coronary care units because of myocardial infarction. Two multimarker panels were applied (Proximity Extension Assay [Olink Bioscience], Multiple Reaction Monitoring mass spectrometry). Lasso analysis (penalized logistic regression), multiple testing-corrected Mann-Whitney tests and Cox regressions were used to assess sex-differences in the concentrations of these biomarkers and their implications on all-cause mortality and major adverse events (median follow-up up to 6.6 years).

**Results:**

Biomarkers provided a very high discrimination between both sexes, when considered simultaneously (c-statistics 0.972). Compared to women, men had higher concentrations of six biomarkers with the most pronounced differences seen for those reflecting atherogenesis, myocardial necrosis and metabolism. Women had higher concentrations of 14 biomarkers with the most pronounced differences seen for those reflecting activation of the renin-angiotensin-aldosterone axis, inflammation and for adipokines. There were no major variations between sexes in the associations of these biomarkers with outcome.

**Conclusions:**

Severable sex-differences exist in the expression of biomarkers in patients with myocardial infarction. While these differences had no impact on outcome, our data suggest the presence of various sex-related pathways involved in the development of coronary atherosclerosis, the progression to plaque rupture and acute myocardial damage, with a greater heterogeneity in women.

## Introduction

Sex-related differences in the pathobiology of acute myocardial infarction (MI) are well established but incompletely understood. This applies to the contribution of cardiovascular risk factors and comorbidities, the severity of coronary artery disease (CAD), the extent of myocardial damage during the ischemic event and pathophysiologic responses thereto. MI in males for example, is commonly caused by atherosclerotic plaque ruptures with occlusive or subocclusive thrombus formation and downstream embolization of thrombotic debris. Women in contrast, more often than men present with MI due to diffuse atherosclerosis, coronary spasm or dissection [1]. This has resulted in a discussion as to whether sex-specific management approaches in MI might be needed [2].

Levels of circulating biomarkers could provide insights into possible sex-related differences in the pathobiology of MI. Recent investigations focusing on the cardiac troponins, B-type natriuretic peptides and C-reactive protein suggested that women have smaller MI compared to men but more pronounced hemodynamic stress and inflammation [3–5]. The pathobiology of MI however, is more complex, and biomarkers reflecting other pathways might provide additional insights.

SWEDEHEART (Swedish Web-system for Enhancement and Development of Evidence-based care in Heart disease Evaluated According to Recommended Therapies) is a nationwide registry prospectively enrolling consecutive patients admitted to Swedish coronary care units or other specialized facilities because of suspected acute coronary syndrome. SWEDEHEART has almost complete nationwide coverage and lifelong follow-up of patients with MI. We realized the potential of a biobank coupled to the registry. In three large centers in Sweden, we developed a blood sampling system based on existing clinical chemistry routines in order to sample patients in the registry in a pragmatic way. Taking advantage of technologies that allow for the simultaneous analysis of large biomarker arrays, we here sought to examine differences in the biomarker profile between men and women with MI. Biomarkers of interest reflected different inflammatory traits, coagulation activity, endothelial dysfunction, atherogenesis, myocardial injury, hemodynamic stress, apoptosis, renal function, glucose- and lipid metabolism. In addition, we aimed at investigating whether sex-differences in these biomarkers were associated with variations in long-term outcome.

## Material and methods

### Study population

This study is part of the TOTAL-AMI (Tailoring Of Treatment in All comers with Acute Myocardial Infarction) project which uses data from SWEDEHEART. The primary aim of TOTAL-AMI is to study the mechanisms and implications of different MI subtypes [6] and of comorbidities (e.g. chronic obstructive pulmonary disease, atrial fibrillation, renal dysfunction) in MI. On admission, patients receive written information about SWEDEHEART, have the right to deny participation and to get their data erased upon request. Written informed consent is not required according to Swedish law.

The study population consists of MI patients admitted between March 2008 and September 2014 at three university hospitals in Stockholm, Lund and Uppsala. The diagnosis of MI was established by the attending physicians at the respective hospitals. In each patient, a fasting plasma sample was obtained at day 1–3 after admission for storage in the SWEDEHEART biobank. This biobank includes three separate biobanks (StockholmHeartBank, LUNDHEARTGENE, Uppsala SWEDEHEART-biobank), located at or in vicinity to the respective hospitals. Written informed consent for biobanking was obtained from all patients.

All data had been made anonymous before the statistical analyses. The study was conducted according to the principles of the 1975 Declaration of Helsinki and had been approved by the Regional Ethical Review Boards in Stockholm, Lund and Uppsala.

### Measurement of circulating biomarkers

Biomarker measurements were performed in plasma ethylenediaminetetraacetic acid samples that had been stored in aliquots at -80°C. Two different methods were used: the commercially available Proximity Extension Assay (PEA [Olink Proseek^®^ Multiplex CVD I^96×96^ kit, Olink Bioscience, Uppsala, Sweden]) and the recently developed Multiple Reaction Monitoring (MRM) mass spectrometry assay panel [7].

The PEA technique measures 92 different biomarkers simultaneously by binding of paired antibodies to target proteins followed by real-time polymerase chain reaction quantification using the Fluidigm BioMark^™^ System [8,9]. This technique has been shown to have a high reproducibility and repeatability with a mean intraassay coefficient of variation of 8% [9]. The MRM assay simultaneously quantifies 87 plasma proteins including three isoforms of apolipoprotein E by the use of nanoscale liquid chromatography coupled to tandem mass spectrometry. Measurements were performed on a TSQ Quantiva mass spectrometer equipped with an EASY-Spray NG ion source and connected to an EASY n-LC 1000 pump (Thermo Scientific, Waltham, MA). The mean intraassay coefficient of variation of this assay is 4.7% [7].

For both methods, biomarkers had been selected a priori based on biological plausibility and clinical relevance in cardiovascular disease. For the purpose of the present analysis, the pathophysiological importance of each individual biomarker has been determined after researches using the Universal Protein Resource [10] and PubMed, see S1 Table. Biomarkers reflecting several or overlapping pathways were assigned the category of best fit.

The results of the PEA analyses are expressed as normalized protein expression units, which are relative log2-transformed concentrations. Protein targets from the MRM assay were quantified using concentration-balanced stable isotope standards and subsequently log2-transformed to enable comparison with the PEA results. Accordingly, biomarker results are reported without standard concentration units. In case of biomarker concentrations below the level of detection, results were replaced by the level of detection value itself. Interleukin-4 was not included in this analysis since this biomarker had very few (3%) results above the level of detection. We considered only total apolipoprotein E but not its isoforms (apolipoprotein E2-E4). For cystatin C, C-reactive protein and adiponectin, two proteins were measured by the MRM assay. In these cases, we used the first biomarker with reported results in the database. This left a total of 175 biomarkers for the analysis.

### Prognostic evaluation

Information on patient outcome was obtained from the mandatory Swedish Patient Registry (hospitalization dates and discharge diagnoses based on International Classification of Diseases, 10^th^ revision, Clinical Modification [ICD-10-CM] codes) and the Swedish Cause of Death Registry, both held by the Swedish Board of Health and Welfare. The outcomes considered for this analysis were all-cause mortality (end of follow-up: May 16^th^, 2018) and major adverse events (MAE; end of follow-up: December 31^st^, 2017), defined as the composite of all-cause mortality, recurrent MI (ICD-10-CM code I21), hospitalization for heart failure (ICD-10-CM code I50) and ischemic stroke (ICD-10-CM code I63). During the first 30 days after the index hospitalization, it is not possible to separate a new MI from the index MI in the Patient Registry. Therefore, only MI occurring 30 days after the index hospitalization were counted.

### Statistical analysis

Given the large number of biomarkers, a rigorous statistical approach was used. First, we calculated the c-statistics as an estimate of the overall discriminative value of biomarkers with respect to sex. For this purpose, we applied Lasso analysis which is a penalized logistic regression model that considers all biomarkers simultaneously in a single calculation. To limit the risk of overfitting and of spurious findings, biomarker concentrations were penalized in the Lasso analyses while covariates were added to adjusted models without transformation. A detailed description is provided in the S1 Methods. The c-statistics was calculated in the total population and in predefined subgroups (age ≥65/< 65 years, diabetes yes/no, estimated glomerular filtration rate <60/≥60 mL/min/1.73m^2^ [Chronic Kidney Disease Epidemiology Collaboration equation], ST-elevation MI yes/no).

Second, Lasso analysis was used to select those biomarkers that discriminated both sexes. An odds ratio >1 corresponds to an increased probability of male sex and an odds ratio <1 corresponds to an increased probability of female sex. Results are reported from crude analyses, in model 1 following adjustment for clinical characteristics (age, hypertension, diabetes, current smoking, renal failure [estimated glomerular filtration rate <60 mL/min/1.73m^2^], previous MI, previous coronary revascularization, previous congestive heart failure, atrial fibrillation on the admission ECG, previous stroke, chronic obstructive pulmonary disease, previous or present cancer and peripheral vascular disease), and in model 2 following additional adjustment for estimates of disease severity (ST-elevation MI, pulmonary rales at admission, cardiogenic shock at admission).

Third, we conducted Mann-Whitney tests to assess sex-related differences in the concentrations of each separate biomarker. For these analyses, correction for multiple testing was made with a permutation-based method which renders adjusted p-values.

Post-hoc sensitivity analyses were performed to assess the discriminative value of biomarkers identified by the Lasso analyses and Mann-Whitney tests in 1) cohorts without and with previously known CAD, defined as a history of coronary revascularization or MI, and 2) when information on admission medications (aspirin, other antiplatelets, oral anticoagulants, betablockers, renin-angiotensin-aldosterone inhibitors, statins) was forced into model 2.

The prognostic implications of biomarkers identified by both the Lasso analysis and Mann-Whitney tests were finally investigated in men and women using Cox regressions with adjustment for clinical characteristics (model 1) and interaction analyses.

Continuous variables are reported as medians with 25^th^ and 75^th^ percentiles, and categoric variables as frequencies and percentages. Missing results were handled using single imputation using age, sex and all biomarkers as predictors, and with logistic regression models for dichotomous results and predictive mean matching for numerical results. For all computations, the software packages R version 3.6.1 (R Foundation for Statistical Computing, Vienna, Austria) and SPSS 24.0 (SPSS Inc., Chicago, IL) were used.

## Results

The population consisted of 856 men and 243 women. ST-elevation was noted upon presentation in 507 (46.3%) patients. Blood samples had been obtained with at day 1 (25^th^, 75^th^ percentiles day 1–2) in both men and women (data available in 801 men and 225 women).

Further details on clinical characteristics are presented in Table 1.

**Table 1:** Clinical characteristics.

	**Men (n = 856)**	**Women (n = 243)**	**Total (n = 1099)**	**Missing data**
**Risk factors**				
Age (years)	64 (57–71)	69 (62–75)	66 (58–72)	-
Current smoking	231 (27.0%)	83 (34.2%)	314 (28.6%)	26 (2.4%)
Hypertension	410 (47.9%)	133 (54.7%)	543 (49.4%)	-
Diabetes	190 (22.2%)	60 (24.7%)	250 (22.7%)	-
Hyperlipidemia	252 (29.4%)	65 (26.7%)	317 (28.8%)	6 (0.5%)
Body mass index (kg/m^2^)	27.0 (24.8–29.4)	25.9 (23.1–29.7)	26.9 (24.6–29.4)	48 (4.4%)
eGFR (mL/min/1.73m^2^)	85.5 (69.2–95.8)	81.9 (63.0–94.1)	84.8 (67.5–95.3)	1 (0.1%)
**History**				
Previous MI	161 (18.9%)	36 (14.9%)	197 (18.0%)	4 (0.4%)
Previous PCI/CABG	168 (19.6%)	26 (10.7%)	194 (17.7%)	-
Previous heart failure	106 (12.4%)	29 (11.9%)	135 (12.3%)	-
Previous stroke	47 (5.5%)	20 (8.2%)	67 (6.1%)	-
Peripheral vascular disease	36 (4.2%)	10 (4.1%)	46 (4.2%)	-
Previous or present cancer	15 (1.8%)	10 (4.1%)	25 (2.3%)	-
COPD	35 (4.1%)	31 (12.8%)	66 (6.0%)	-
**Medication at admission**				
Aspirin	235 (27.6%)	67 (27.7%)	302 (27.6%)	6 (0.5%)
P2Y12 blockers	55 (6.5%)	10 (4.1%)	65 (6.0%)	8 (0.7%)
Oral anticoagulants	32 (3.8%)	8 (3.3%)	40 (3.7%)	6 (0.5%)
Betablockers	255 (30.2%)	89 (36.8%)	344 (31.7%)	13 (1.2%)
RAAS-inhibitors	254 (30.1%)	77 (31.8%)	331 (30.5%)	12 (1.1%)
Statins	243 (28.6%)	64 (26.4%)	307 (28.1%)	7 (0.6%)
**Findings upon presentation**				
Sinus rhythm	787 (92.3%)	226 (93.0%)	1013 (92.4%)	1 (0.1%)
Atrial fibrillation	46 (5.4%)	14 (5.8%)	60 (5.5%)	1 (0.1%)
ST-depression	131 (15.4%)	36 (14.8%)	167 (15.2%)	3 (0.3%)
ST-elevation	384 (45.0%)	123 (50.6%)	507 (46.3%)	14 (1.3%)
Pulmonary rales	46 (5.5%)	21 (8.9%)	67 (6.2%)	21 (1.9%)
Cardiogenic shock	4 (0.5%)	1 (0.4%)	5 (0.5%)	13 (1.2%)
	**Men (n = 856)**	**Women (n = 243)**	**Total (n = 1099)**	**Missing data**
**Angiographic findings** *				
not evaluable	23 (2.7%)	7 (2.9%)	30 (2.7%)	-
normal/non-occlusive	33 (3.9%)	25 (10.3%)	58 (5.3%)	-
1–2 vessel disease	592 (69.2%)	174 (71.6%)	766 (69.7%)	-
3 vessel disease	208 (24.3%)	37 (15.2%)	245 (22.3%)	-
**Echocardiography** †				
LVEF ≥0.50	134 (62.0%)	468 (60.4%)	602 (60.7%)	-
LVEF 0.40–0.49	42 (19.4%)	152 (19.6%)	194 (19.5%)	-
LVEF 0.30–0.39	27 (12.5%)	119 (15.4%)	146 (14.7%)	-
LVEF <0.30	13 (6.0%)	36 (4.6%)	49 (4.9%)	-
**In-hospital coronary revascularization**	770 (90.0%)	202 (83.1%)	972 (88.4%)	-

*n = = 1069.

^†^ n = 991.

Data are presented as numbers (with percentages) or medians (with 25^th^, 75^th^ percentiles).

eGFR: Estimated glomerular filtration rate; MI: Myocardial infarction; PCI: Percutaneous coronary intervention; CABG: Coronary artery bypass grafting; COPD: Chronic obstructive pulmonary disease; RAAS: Renin-angiotensin-aldosterone system; LVEF: Left-ventricular ejection fraction.

The Lasso analysis yielded a very high discriminative value of biomarkers overall with respect to sex. The c-statistics was 0.972 in the crude analysis and remained largely unchanged following adjustment for clinical characteristics (c-statistics: 0.973; model 1) and estimates of disease severity (c-statistics 0.969; model 2). Even in the prespecified subgroups, the non-adjusted adjusted c-statistics were >0.910 and remained high in the adjusted models (S2 Table).

Thirty-four biomarkers provided consistent discriminatory value both in the crude Lasso analysis, following adjustment for clinical characteristics (model 1) and additionally for estimates of disease severity (model 2), see S3 Table. Eighteen biomarkers discriminated men from women and 16 biomarkers discriminated women from men.

**Fig 1:**
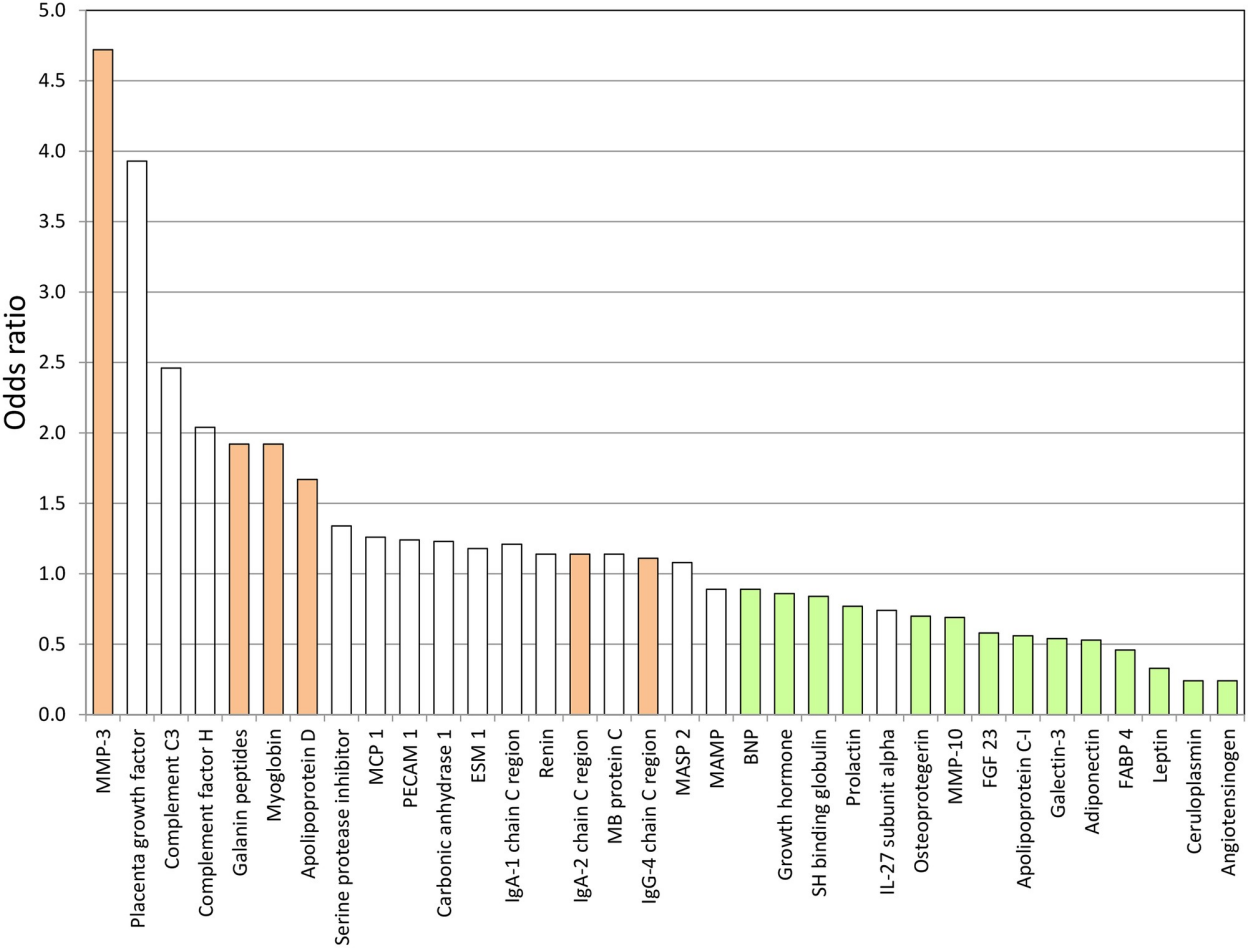
Biomarkers with discriminatory value regarding sex according to results from the Lasso analysis. The red-shaded and green-shaded columns indicate biomarkers with higher concentrations in men (n = 6) and women (n = 14), respectively, according to Mann-Whitney tests with correction for multiple testing. MMP-3: Matrix metalloproteinase-3; MCP 1: Monocyte chemotactic protein 1; PECAM 1: Platelet endothelial cell adhesion molecule 1; ESM-1: Endothelial cell-specific molecule-1; MB: Mannose-binding; MASP 2: Mannan-binding lectin serine protease 2; MAMP: Membrane-bound aminopeptidase P; BNP: B-type natriuretic peptide; SH: Sex hormone; IL: Interleukin; FGF 23: Fibroblast growth factor 23; FABP 4: Fatty acid-binding protein 4.

Using Mann-Whitney tests with correction for multiple testing, the concentrations of six “male” and 14 “female” biomarkers differed significantly (p<0.05), see Fig 1 and S1 Fig, and the present investigation will focus on these. The findings from the Lasso analysis together with the concentrations of these biomarkers and information on their biological function is presented in Table 2. The distributions of biomarker concentrations are depicted in empirical cumulative distribution function plots presented in S2 and S3 Figs.

**Table 2:** Biomarker concentrations and results from the Lasso analysis. A) Biomarkers with higher concentrations in men; B) Biomarkers with higher concentrations in women.

**A)**		**Biomarker concentrations (log2-transformed)**			**Odds ratio (Lasso analysis)**		
**Biomarker**	**Pathobiological importance**	**Men**	**Women**	**p-value**	**Crude**	**Model 1**	**Model 2**
MMP-3	Atherogenesis	1.92 (-0.07–5.32)	1.41 (-0.06–3.90)	<0.001	3.53	4.39	4.72
Galanin peptides	Glucose metabolism	5.29 (3.05–7.12)	4.82 (2.76–7.41)	<0.001	1.91	1.91	1.92
Myoglobin	Myocardial damage	6.55 (4.78–8.99)	6.37 (4.22–8.66)	0.021	1.56	1.79	1.92
Apolipoprotein D	Lipid metabolism	6.11 (3.05–8.35)	5.90 (4.15–7.84)	0.041	1.31	1.67	1.67
IgA-2 chain C region	Pro-inflammatory	12.1 (0.6–15.1)	11.8 (8.4–14.3)	<0.001	1.09	1.14	1.14
IgG-4 chain C region	Pro-inflammatory	8.19 (-0.15–12.67)	7.70 (0.83–11.29)	<0.001	1.10	1.11	1.11
**B)**		**Biomarker concentrations (log2-transformed)**			**Odds ratio (Lasso analysis)**		
**Biomarker**	**Pathobiological importance**	**Men**	**Women**	**p-value**	**Crude**	**Model 1**	**Model 2**
Angiotensinogen	RAAS-axis	6.54 (3.53–8.27)	6.75 (5.85–9.46)	<0.001	0.34	0.25	0.24
Ceruloplasmin	Acute phase reactant	7.58 (4.89–9.29)	7.83 (6.49–9.64)	<0.001	0.36	0.27	0.24
Leptin	Adipokine	5.04 (1.02–7.63)	6.03 (1.78–8.01)	<0.001	0.39	0.34	0.33
FABP 4	Pro-inflammatory	2.60 (1.15–5.30)	3.18 (1.34–5.63)	<0.001	0.52	0.48	0.46
Adiponectin	Adipokine	-0.10 (-3.09–2.79)	0.44 (-2.44–2.97)	<0.001	0.54	0.55	0.53
Galectin-3	Myocardial function	4.79 (3.36–6.42)	4.97 (3.54–6.32)	<0.001	0.65	0.53	0.54
Osteoprotegerin	Atherogenesis	9.16 (7.77–11.52)	9.34 (7.65–11.15)	<0.001	0.67	0.74	0.70
FGF 23	Hormone	3.12 (1.23–9.48)	3.43 (1.97–9.54)	<0.001	0.73	0.61	0.58
Apolipoprotein C-I	Lipid metabolism	5.18 (1.89–7.02)	5.34 (3.71–6.92)	<0.001	0.75	0.57	0.56
MMP-10	Atherogenesis	7.28 (5.09–10.77)	7.47 (6.16–9.36)	0.010	0.77	0.70	0.69
Growth hormone	Hormone	6.87 (2.82–11.04)	7.51 (3.80–10.61)	<0.001	0.83	0.85	0.86
SH binding globulin	Hormone	2.46 (-2.13–6.43)	2.96 (-0.44–6.29)	<0.001	0.85	0.86	0.84
Prolactin	Angiogenesis	4.01 (1.68–7.03)	4.27 (2.00–6.50)	0.008	0.89	0.77	0.77
BNP	Myocardial function	2.66 (0.67–7.44)	3.45 (0.67–7.70)	<0.001	0.90	0.88	0.89

Only biomarkers with discriminatory value regarding sex (Lasso analysis) and significant sex-differences in concentrations (Mann-Whitney test) are listed.

Model 1: Adjusted for age, hypertension, diabetes, current smoking, renal failure, previous myocardial infarction, previous coronary revascularization, previous congestive heart failure, atrial fibrillation on the admission ECG, previous stroke, chronic obstructive pulmonary disease, previous or present cancer and peripheral vascular disease.

Model 2: Additionally adjusted for ST-elevation myocardial infarction, pulmonary rales at admission and cardiogenic shock at admission.

OR >1 correspond to an increased probability of male sex. OR <1 correspond to an increased probability of female sex.

MMP: Matrix metalloproteinase; Ig: Immunoglobulin; RAAS: Renin-angiotensin-aldosterone; FABP 4: Fatty-acid binding protein 4; FGF 23: Fibroblast growth factor 23; SH: Sex hormone; BNP: B-type natriuretic peptide.

Almost all of the 20 biomarkers also discriminated both sexes in cohorts without and with previously known CAD (S4 Table). Interestingly, some cardiac biomarkers (i.e. myoglobin, B-type natriuretic peptide, galectin-3, fibroblast growth factor 23) tended to provide weaker or no discriminative value in patients with previously known CAD. Additional adjustment of model 2 for discharge medications did not alter the overall findings substantially (S5 Table).

During a median follow-up of 6.6. years, 131 (15.3%) men and 52 (21.4%) women died (p = 0.031). During a median follow-up of 5.7 years, 276 (32.2%) men suffered a MAE (in total 120 deaths, 119 recurrent MI, 43 strokes and 89 hospitalizations for heart failure). Seventy-five (30.9%) women suffered a MAE (in total 51 deaths, 30 recurrent MI, 12 strokes and 24 hospitalizations for heart failure); p = 0.698 for comparison with men. Table 3 presents the hazard ratios with adjustment for clinical characteristics according to model 1. Despite gradually different point estimates of the hazard ratios, interaction analysis indicated that almost all biomarkers were similarly predictive in men and women. An interaction of sex on the association with MAE emerged only for leptin concentrations, albeit at borderline levels of significance (p = 0.040).

**Table 3:** Associations of biomarker concentrations with outcome. A) All-cause mortality; B) Major adverse events.

**A)**	**Hazard ratio (95% confidence interval)**		
**Biomarkers with higher concentrations in men**	**Men**	**Women**	**p int**.
MMP-3	1.60 (1.28–1.98)	2.05 (1.42–2.96)	0.364
Galanin peptides	0.79 (0.60–1.02)	0.67 (0.45–1.00)	0.878
Myoglobin	1.33 (1.08–1.63)	1.26 (0.91–1.76)	0.445
Apolipoprotein D	1.11 (0.84–1.46)	1.02 (0.63–1.66)	0.962
IgA-2 chain C region	0.98 (0.84–1.15)	1.08 (0.82–1.42)	0.520
IgG-4 chain C region	0.89 (0.80–0.99)	0.89 (0.75–1.05)	0.544
**Biomarkers with higher concentrations in women**			
Angiotensinogen	1.25 (0.86–1.81)	1.64 (0.98–2.74)	0.358
Ceruloplasmin	1.31 (0.90–1.92)	1.63 (0.93–2.85)	0.589
Leptin	0.84 (0.71–1.00)	0.76 (0.57–1.00)	0.543
FABP 4	1.57 (1.19–2.09)	1.21 (0.80–1.82)	0.146
Adiponectin	1.27 (1.00–1.62)	1.24 (0.86–1.79)	0.744
Galectin-3	1.21 (0.84–1.75)	0.80 (0.43–1.49)	0.280
Osteoprotegerin	1.64 (1.14–2.38)	1.71 (0.88–3.35)	0.676
FGF 23	1.56 (1.31–1.87)	1.59 (1.31–1.92)	0.788
Apolipoprotein C-I	0.75 (0.54–1.04)	0.74 (0.42–1.32)	0.688
MMP-10	1.41 (1.07–1.84)	1.61 (0.98–2.65)	0.974
Growth hormone	1.17 (1.05–1.31)	1.16 (0.96–1.41)	0.955
SH binding globulin	1.05 (0.90–1.23)	1.32 (1.02–1.71)	0.149
Prolactin	1.22 (0.96–1.57)	1.07 (0.75–1.53)	0.697
BNP	1.21 (1.08–1.36)	1.60 (1.31–1.96)	0.067
**B)**	**Hazard ratio (95% confidence interval)**		
**Biomarkers with higher concentrations in men**	**Men**	**Women**	**p int**.
MMP-3	1.46 (1.24–1.70)	1.49 (1.07–2.07)	0.812
Galanin peptides	0.91 (0.76–1.08)	0.84 (0.60–1.17)	0.986
Myoglobin	1.25 (1.09–1.44)	1.19 (0.90–1.56)	0.620
Apolipoprotein D	1.12 (0.93–1.34)	0.98 (0.67–1.43)	0.611
IgA-2 chain C region	1.01 (0.91–1.12)	1.01 (0.86–1.34)	0.657
IgG-4 chain C region	0.91 (0.85–0.98)	0.95 (0.83–1.10)	0.411
**Biomarkers with higher concentrations in women**			
Angiotensinogen	1.24 (0.97–1.60)	1.17 (0.75–1.83)	0.935
Ceruloplasmin	1.38 (1.07–1.78)	1.36 (0.85–2.17)	0.903
Leptin	0.98 (0.86–1.13)	0.71 (0.56–0.90)	0.040
FABP 4	1.35 (1.14–1.63)	0.91 (0.65–1.28)	0.099
Adiponectin	1.12 (0.95–1.31)	1.08 (0.81–1.43)	0.792
Galectin-3	1.22 (0.95–1.56)	0.72 (0.43–1.19)	0.121
Osteoprotegerin	1.65 (1.26–2.16)	1.38 (0.80–2.36)	0.632
FGF 23	1.39 (1.21–1.60)	1.40 (1.19–1.65)	0.998
Apolipoprotein C-I	0.96 (0.77–1.19)	0.83 (0.52–1.32)	0.681
MMP-10	1.16 (0.97–1.40)	1.45 (1.00–2.09)	0.359
Growth hormone	1.15 (1.07–1.23)	1.09 (0.95–1.26)	0.683
SH binding globulin	1.02 (0.92–1.14)	1.04 (0.85–1.27)	0.853
Prolactin	1.08 (0.91–1.28)	0.94 (0.69–1.26)	0.481
BNP	1.19 (1.10–1.29)	1.34 (1.15–1.56)	0.161

Analyses adjusted for age, hypertension, diabetes, current smoking, renal failure, previous myocardial infarction, previous coronary revascularization, previous congestive heart failure, atrial fibrillation on the admission ECG, previous stroke, chronic obstructive pulmonary disease, previous or present cancer and peripheral vascular disease.

P int. refers to the interaction of sex on the association of each respective biomarker with the respective outcome.

MMP: Matrix metalloproteinase; Ig: Immunoglobulin; FABP 4: Fatty-acid binding protein 4; FGF 23: Fibroblast growth factor 23; SH: Sex hormone; BNP: B-type natriuretic peptide.

## Discussion

In this study, we investigated sex-related differences in the pathobiology of MI by measuring two large arrays of cardiovascular biomarkers. We used newer technologies which allowed us to simultaneously interrogate multiple pathways of clinical importance.

Already in the first step of our analyses we found a high discriminative value of biomarkers overall with respect to sex, consistent with a differential expression in men and women. The c-statistics was >0.970 in the total cohort and remained high in prespecified subgroups, even when taking clinical characteristics and estimates of disease severity into consideration.

Certain biomarkers revealed particularly strong discriminative value, highlighting potential sex-specific differences in pathways that are relevant in stable CAD and MI. Men had higher concentrations of biomarkers reflecting cardiomyocyte necrosis (i.e. myoglobin) and the promotion of atherosclerosis (i.e. matrix metalloproteinase-3). This corroborates with results from previous investigations demonstrating greater myocardial damage and more severe CAD in men with MI [1,3–5]. Even some biomarkers of glucose- and lipid metabolism (i.e. galanin peptides, apolipoprotein D) discriminated men from women. We assume that this reflects a male-specific disposition for metabolic traits involved in the development of CAD.

In women, higher concentrations were noted for a larger number of biomarkers compared to men. These “female” biomarkers reflected various pathobiological aspects in MI, i.e. inflammation, atherogenesis and myocardial dysfunction, but also glucose- and lipid metabolism. The difference to men was most pronounced for angiotensinogen and adipokines (i.e. leptin, adiponectin). With respect to angiotensinogen, our data point towards an upregulation of the renin-angiotensin-aldosterone axis in women with MI even when controlling for the presence of hypertension and medications upon admission. The finding of higher adipokine concentrations in women with MI is consistent with data from studies in community-dwelling subjects [11,12] and patients with acute coronary syndrome [13,14], and indicate that adiposity pathways are more relevant for the development of atherosclerosis in women than in men [15,16].

Female sex is an important modulator of both innate and adaptive immunity. Sex-differences in the inflammatory immune response during the development of atherosclerosis have been suggested but remain controversial [17]. Still, women from our cohort had higher concentrations of several biomarkers involved in inflammatory processes, i.e. ceruloplasmin, osteoprotegerin, matrix metalloproteinase-10, prolactin and fatty acid binding protein-4, a protein exerting both adipokine-like and proinflammatory effects.

Women also had higher concentrations of some cardiac biomarkers, i.e. galectin-3 and B-type natriuretic peptide. Galectin-3 is involved in cardiac fibrosis and remodeling, and our finding may have some bearing on the presence of diastolic dysfunction, known to be more prevalent in women than in men [18]. B-type natriuretic peptide reflects hemodynamic stress and ischemia. However, even in apparently healthy subjects, levels tend to be higher in women which has been ascribed the effect of sex hormones [19]. Women also had higher concentrations of fibroblast growth factor 23. While regulating phosphate metabolism under physiologic conditions, this hormone is also produced in cardiac fibroblasts during MI and is involved in adverse myocardial remodeling [20,21].

Interestingly, the discriminative value of some cardiac biomarkers was smaller in patients with previously known CAD and became non-existent in the case of myoglobin and B-type natriuretic peptide. This suggests that once that CAD has developed, sex-differences in pathobiological mechanisms and responses to the ischemic event become less pronounced.

Our findings lead to the question whether the observed biomarker differences represent sex-specific variations in the pathophysiologic response to an MI or intrinsic differences between the sexes. For all biomarkers with exception for osteoprotegerin [11,22], the distribution pattern in our cohort of MI patients was similar as seen in general population studies, see S6 Table. Changes in acute MI have been described only for a few biomarkers [23–25], and as to whether inherent sex-specific differences in biomarker concentrations may be amplified during an ischemic event together with the consequences thereof remains to be studied.

What are the clinical implications of our findings? Our results support the presence of sex-differences in the pathobiology of MI with more pathways being involved in women compared to men. How these differences in turn relate to the different facets of MI which are overrepresented in women, needs further investigation. The sex-differences in the biomarker profile appeared on the other hand, not to be associated with variations in outcome. Interaction analysis yielded similar prognostic value for almost all biomarkers in men and women. The only exception was leptin for which higher concentrations predicted lower MAE risk in women with a weak, albeit significant interaction of sex. Although regarded as a proinflammatory adipokine, it has been postulated that leptin also might exert protective effects in patients with established CAD [26]. However, results from previous investigations are conflicting in this regard [11,13]. Whether biomarker-guided sex-differentiated management routines might have an impact on improving outcome in men and women with MI remains thus, to be elucidated.

There are some limitations to our study that need to be considered. Although all hospitals participating in SWEDEHEART are annually monitored, the data cannot be of the same quality as in a prospective observational study. However, the agreement between the information entered in the registry and the medical records is around 96% [27]. While the SWEDEHEART framework recommends the use of criteria outlined in the Universal Definition for the diagnosis of MI [6,28], there was no independent adjudication of the index diagnosis. This implies some risk of misclassification. The type of MI per Universal Definition [6] had not been consistently documented during the observation time. Compared to proteomic approaches, our results are not based on an unbiased sampling strategy. Instead, candidate biomarkers had been selected by the developers of the PEA- and MRM techniques. We cannot comment on the potential implications of cardiac troponin in the context of our analysis since this biomarker is not included as analyte in the PEA- and MRM-panels. Moreover, available cardiac troponin data in SWEDEHEART had been obtained with the use of different assays with different sensitivities and variances (S7 Table), resulting in heterogeneity that hardly can be handled in the analyses. The PEA technique does not provide standard concentration units which makes comparisons with clinically applied cut-offs difficult. We lack a control group of healthy subjects and can thus, not comment on changes in biomarker levels during MI. The timepoint of sampling is given per days in the database why we are unable to comment on the impact of very early biomarker kinetics on our findings. Some biomarkers are known to be influenced in women by menopausal or hormone replacement status. Such information is not collected in SWEDEHEART. However, with a median age of 68 years in women, it is unlikely that variations in hormone status would exhibit strong associations with biomarker concentrations. Indexes of adipose tissue distribution are not systematically documented in SWEDEHEART apart from the body mass index. For this reason, we are not able to further explore the sex-specific interrelations of adipokines, obesity and outcome. Since our study patients had been admitted to the coronary care units of three university hospitals, some selection bias may be present. Given the observational nature of our study, our results finally should be regarded as hypothesis-generating and warrant further investigation.

In conclusion, several differences exist in the sex-specific expression of circulating biomarkers in MI with more pathways being involved in women. Our findings appear rather to reflect variations in the contribution of these pathways to the development of CAD than variations in the response to the ischemic event. Consequences of sex-specific variations on outcome were very limited. For our future evaluations of MI patients, it is thus, assuring that the prospective values of biomarkers were overall similar between the sexes. Nonetheless, a more complete understanding of sex-differences in the pathobiology of MI is needed.

## Supporting information

S1 Fig Results from the Mann-Whitney tests. (DOCX)

S2 Fig Empirical cumulative distribution plots. Biomarkers with higher concentrations in men.
(DOCX)

S3 Fig Empirical cumulative distribution plots. Biomarkers with higher concentrations in women.
(DOCX)

S1 Table Biomarkers analyzed by the Proseek Multiplex CVD I and Multiple Reaction Monitoring platforms and their suggested pathobiological importance in cardiovascular disease. (DOCX)

S2 Table Discriminative value of biomarkers overall with respect to sex in the total population and predefined subgroups. (DOCX)

S3 Table Results from the Lasso analysis. A) Biomarkers with higher concentrations in men; B) Biomarkers with higher concentrations in women.
(DOCX)

S4 Table Results from the Lasso analysis in subcohorts without and with previously known coronary artery disease. A) Biomarkers with higher concentrations in men; B) Biomarkers with higher concentrations in women.
(DOCX)

S5 Table Results from the Lasso analysis following additional adjustment of model 2 for medications at admission. A) Biomarkers with higher concentrations in men; B) Biomarkers with higher concentrations in women.
(DOCX)

S6 Table Summary of selected studies investigating the associations of biomarker levels with sex, changes during myocardial infarction and sex-related differences in outcome. (DOCX)

S7 Table Cardiac troponin assays used during the study period. (DOCX)

S1 Methods Description of the Lasso analysis. (DOCX)
