# Supplementary material for: Sex-differences in circulating biomarkers during acute myocardial infarction: An analysis from the SWEDEHEART registry
Source: PLoS One. 2021 Apr 8;16(4):e0249830. doi: 10.1371/journal.pone.0249830 (PMC8031406; doi:10.1371/journal.pone.0249830)
Supplement: S1 Fig — (DOCX) [file pone.0249830.s001.docx]

**S1 Figure. Results from the Mann-Whitney tests.**

**
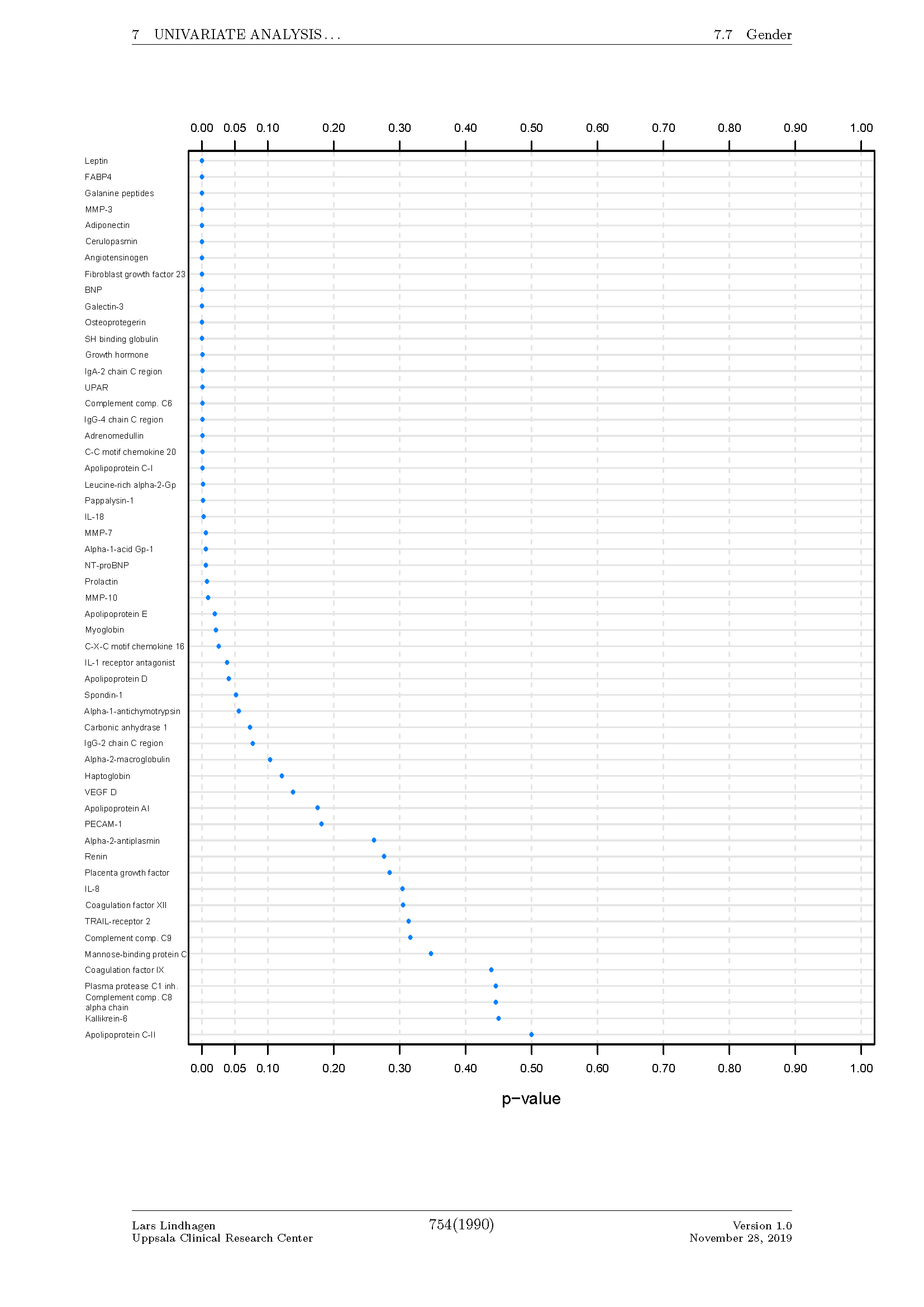
**

P-values are adjusted following correction for multiple testing.

FABP 4: Fatty acid-binding protein 4; MMP: Matrix metalloproteinase; BNP: B-type natriuretic peptide; SH: Sex hormone; Ig: Immunoglobulin; UPAR: Urokinase plasminogen activator surface receptor; Comp: Component; Gp: Glykoprotein; IL: Interleukin; NT-proBNP: N-terminal pro-B-type natriuretic peptide; VEGF D: Vascular endothelial growth factor D; PECAM: PECAM 1: Platelet endothelial cell adhesion molecule 1; TRAIL: TNF-related apoptosis-inducing ligand; Inh: Inhibitor;
