# Supplementary material for: Sex-differences in circulating biomarkers during acute myocardial infarction: An analysis from the SWEDEHEART registry
Source: PLoS One. 2021 Apr 8;16(4):e0249830. doi: 10.1371/journal.pone.0249830 (PMC8031406; doi:10.1371/journal.pone.0249830)
Supplement: S2 Fig — Biomarkers with higher concentrations in men. (DOCX) [file pone.0249830.s002.docx]

**S2 Figure. Empirical cumulative distribution plots. Biomarkers with higher concentrations in men.**

| 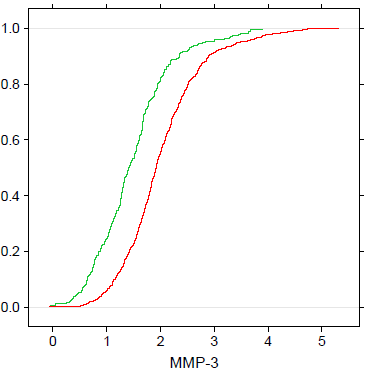 |  |
| --- | --- |
|  |  |
|  |  |

Biomarker concentrations are based on log2-transfomred results. Red lines represent biomarker concentrations in men, green lines represent biomarker concentrations in women.

MMP: Matrix metalloproteinase; Ig: Immunoglobulin.
