# Supplementary material for: Sex-differences in circulating biomarkers during acute myocardial infarction: An analysis from the SWEDEHEART registry
Source: PLoS One. 2021 Apr 8;16(4):e0249830. doi: 10.1371/journal.pone.0249830 (PMC8031406; doi:10.1371/journal.pone.0249830)
Supplement: S1 Table — (DOCX) [file pone.0249830.s004.docx]

**S1 Table. Biomarkers analyzed by the Proseek Multiplex CVD I and Multiple Reaction Monitoring platforms and their suggested pathobiological importance in cardiovascular disease.**

|  |  |
| --- | --- |
| **Proseek Multiplex CVD I** | **Multiple Reaction Monitoring assay** |
|  |  |
|  |  |
| **Pro-inflammatory biomarkers** | |
|  |  |
| C-C motif chemokine 3 | CD5 antigen-like |
| C-C motif chemokine 4 | Complement C1q subcomponent subunit A |
| C-C motif chemokine 20 | Complement C1q subcomponent subunit B |
| Chitinase-3-like protein 1 | Complement C1q subcomponent subunit C |
| C-X-C motife chemokine 1 | Complement C1r subcomponent |
| C-X-C motif chemokine 6 | Complement C1s subcomponent |
| C-X-C motif chemokine 16 | Complement C2 |
| Eosinophil cationic protein | Complement C3 |
| Fatty-acid binding protein 4 | Complement C4-B |
| Follistatin | Complement C5 |
| Fractalkine | Complement component C6 |
| Interleukin-4 | Complement component C7 |
| Interleukin-6 | Complement component C8 alpha chain |
| Interleukin-6 receptor subunit alpha | Complement component C8 beta chain |
| Interleukin-8 | Complement component C8 gamma chain |
| Interleukin-16 | Complement component C9 |
| Interleukin-18 | Complement factor B |
| Interleukin-27 subunit alpha | IgA-1 chain C region |
| Macrophage colony-stimulating factor 1 | IgA-2 chain C region |
| NF-kappa-B essential modulator | IgG-1 chain C region |
| CA 125 | IgG-2 chain C region |
| Pentraxin 3 | IgG-3 chain C region |
| Protein S100-A12 | IgG-4 chain C region |
| Proto-oncogene tyrosine-protein kinase Src | IgK chain C region |
| P-selectin glycoprotein ligand 1 | IgM chain C region |
| Receptor for AGE | L-selectin |
| Stem cell factor | Lipopolysaccharide-binding protein |
| TNF-related activation-induced cytokine | Mannan-binding lectin serine protease 1 |
| TNF-receptor superfamily member 5 | Mannan-binding lectin serine protease 2 |
| TNF-ligand superfamily member 14 | N-acetylmuramoyl-L-alanine amidase |
|  | Properdin |
|  | |
|  | |
| **Anti-inflammatory biomarkers** | |
|  | |
| Growth differentiation factor-15 | Complement factor I |
| Interleukin-1 receptor antagonist protein | Complement factor H |
|  | Complement decay-accelerating factor |
|  | |
|  | |
| **Proseek Multiplex CVD I** | **Multiple Reaction Monitoring assay** |
|  | |
|  | |
| **Acute phase reactants** | |
|  | |
|  | Alpha-1-acid glycoprotein 1 |
|  | Alpha-1-antichymotrypsin |
|  | Alpha-1-antitrypsin |
|  | C-reactive protein |
|  | Ceruloplasmin |
|  | Mannose-binding protein C |
|  | Lipopolysaccharide-binding protein |
|  | Plasma protease C1 inhibitor |
|  | Serum amyloid A-1 protein |
|  | Serum amyloid A-2 protein |
|  | |
|  | |
| **Pro-coagulatory biomarkers** | |
|  | |
| Tissue factor | Alpha-2-antiplasmin |
| CD40 ligand | Alpha-2-macroglobulin |
| Proteinase-activated receptor 1 | Coagulation factor IX |
|  | Coagulation factor V |
|  | Coagulation factor X |
|  | Coagulation factor XI |
|  | Coagulation factor XII |
|  | Coagulation factor XIII A chain |
|  | Coagulation factor XIII B chain |
|  | Fibrinogen alpha chain |
|  | Fibrinogen beta chain |
|  | Fibrinogen gamma chain |
|  | Prothrombin |
|  | Serine protease inhibitor |
|  | Thrombospondin-1 |
|  | Vitamin K-dependent protein Z |
|  | von Willebrand factor |
|  | |
|  | |
| **Anti-coagulatory biomarkers** | |
|  | |
| Thrombomodulin | Antithrombin III |
| Tissue-type plasminogen activator | Beta-2-glycoprotein 1 |
| uPAR | Plasma protease C1 inhibitor |
|  | Plasminogen |
|  | Tetranectin |
|  | Tissue factor pathway inhibitor |
|  | Vitamin K-dependent protein C |
|  | Vitamin K-dependent protein S |
|  | |
|  | |
| **Biomarkers of endothelial function** | |
|  | |
| Adrenomedullin |  |
| E-selectin |  |
|  | |
|  | |
| **Biomarkers of the renin-angiotensin-aldosterone axis** | |
|  | |
| Renin | Angiotensinogen |
|  | |
|  | |
| **Proseek Multiplex CVD I** | **Multiple Reaction Monitoring assay** |
|  | |
|  | |
| **Biomarkers of angiogenesis** | |
|  | |
| Angiopoietin-1 receptor | Leucine-rich alpha-2-glycoprotein |
| Beta-nerve growth factor |  |
| Endothelial cell-specific molecule 1 |  |
| Platelet-derived growth factor subunit B |  |
| Prolactin |  |
| Vascular endothelial growth factor A |  |
| Vascular endothelial growth factor D |  |
|  | |
|  | |
| **Atherogenic biomarkers** | |
|  | |
| Cathepsin D |  |
| Cathepsin L1 |  |
| Cystatin-B |  |
| Heat shock 27 kDa protein |  |
| Kallikrein-6 |  |
| Kallikrein-11 |  |
| Lectin-like oxidized LDL receptor 1 |  |
| Matrix metalloproteinase-1 |  |
| Matrix metalloproteinase-3 |  |
| Matrix metalloproteinase-7 |  |
| Matrix metalloproteinase-10 |  |
| Matrix metalloproteinase-12 |  |
| Membrane-bound aminopeptidase P |  |
| Monocyte chemotactic protein 1 |  |
| Myeloperoxidase |  |
| Osteoprotegerin |  |
| Pappalysin-1 |  |
| Placenta growth factor |  |
| Platelet endothelial cell adhesion molecule |  |
|  | |
|  | |
| **Biomarkers of myocardial function and damage** | |
|  | |
| B-type natriuretic peptide | Carbonic anhydrase 1 |
| Galectin-3 | Fibronectin |
| Melusin |  |
| Myoglobin |  |
| N-terminal pro-B-type natriuretic peptide |  |
| ST2 protein |  |
|  | |
|  | |
| **Biomarkers of apoptosis** | |
|  | |
| Caspase-8 | Clusterin |
| TNF-related apoptosis-inducing ligand |  |
| TNF-related apoptosis-inducing ligand receptor 2 |  |
| TNF-receptor 1 |  |
| TNF-receptor 2 |  |
| TNF-receptor superfamily member 6 |  |
|  | |
|  | |
| **Biomarkers of glucose metabolism** | |
|  | |
| Galanin peptides |  |
| SIR2-like protein |  |
|  | |
|  | |
| **Proseek Multiplex CVD I** | **Multiple Reaction Monitoring assay** |
|  | |
|  | |
| **Biomarkers of lipid metabolism** | |
|  | |
|  | Apolipoprotein A-I |
|  | Apolipoprotein A-II |
|  | Apolipoprotein A-IV |
|  | Apolipoprotein B-100 |
|  | Apolipoprotein C-I |
|  | Apolipoprotein C-II |
|  | Apolipoprotein C-III |
|  | Apolipoprotein D |
|  | Apolipoprotein E |
|  | Apolipoprotein E2 isoform |
|  | Apolipoprotein E3 isoform |
|  | Apolipoprotein E4 isoform |
|  | Apolipoprotein E phenotype |
|  | Apolipoprotein L1 |
|  | Apolipoprotein M |
|  | |
|  | |
| **Adipokines** | |
|  | |
| Leptin | Adiponectin |
| Resistin |  |
|  | |
|  | |
| **Other biomarkers of metabolism** | |
|  |  |
| Agouti-related protein | Haptoglobin |
|  | Hemopexin |
|  | |
|  | |
| **Growth hormones and biomarkers involved in endocrine regulation** | |
|  | |
| Dickkopf-related protein | Sex hormone binding globulin |
| Epidermal growth factor |  |
| Fibroblast growth factor 23 |  |
| Growth hormone |  |
| Heparin-binding EGF-like growth factor |  |
| Hepatocyte growth factor |  |
| Spondin-1 |  |
|  | |
|  | |
| **Biomarkers of renal function** | |
|  | |
| TIM-1 | Cystatin C |
|  |  |

CA 125: Ovarian cancer-related tumor marker CA 125; Ig: Immunoglobulin; AGE: advanced glycosylation end products; TNF: tumor necrosis factor; uPAR: Urokinase plasminogen activator surface receptor; TIM-1: T-cell immunoglobulin and mucin domain 1.
