# Supplementary material for: Sex-differences in circulating biomarkers during acute myocardial infarction: An analysis from the SWEDEHEART registry
Source: PLoS One. 2021 Apr 8;16(4):e0249830. doi: 10.1371/journal.pone.0249830 (PMC8031406; doi:10.1371/journal.pone.0249830)
Supplement: S2 Table — (DOCX) [file pone.0249830.s005.docx]

**S2 Table. Discriminative value of biomarkers overall with respect to sex in the total population and predefined subgroups.**

|  |  |  |  |
| --- | --- | --- | --- |
|  | **C-statistics** | | |
|  |  |  |  |
|  |  |  |  |
|  | **Crude** | **Model 1** | **Model 2** |
|  |  |  |  |
|  |  |  |  |
| All patients | 0.972 | 0.973 | 0.969 |
|  |  |  |  |
| Age ≥65 years | 0.950 | 0.942 | 0.933 |
| Age <65 years | 0.988 | 0.973 | 0.973 |
|  |  |  |  |
| Diabetes | 0.917 | 0.884 | 0.888 |
| No diabetes | 0.974 | 0.970 | 0.968 |
|  |  |  |  |
| eGFR <60 mL/min/1.73m^2^ | 0.923 | 0.877 | 0.859 |
| eGFR ≥60 mL/min/1.73m^2^ | 0.971 | 0.965 | 0.963 |
|  |  |  |  |
| STEMI | 0.975 | 0.972 | 0.970 |
| NSTEMI | 0.949 | 0.956 | 0.954 |
|  |  |  |  |

Model 1: adjusted for age, hypertension, diabetes, current smoking, renal failure, previous MI, previous coronary revascularization, previous congestive heart failure, atrial fibrillation on the admission ECG, previous stroke, chronic obstructive pulmonary disease, dementia, previous or present cancer and peripheral vascular disease.

Model 2: additionally adjusted for STEMI, pulmonary rales at admission, cardiogenic shock at admission.

eGFR: estimated glomerular filtration rate; STEMI: ST-elevation myocardial infarction; NSTEMI: non ST-elevation myocardial infarction.
