# Supplementary material for: Sex-differences in circulating biomarkers during acute myocardial infarction: An analysis from the SWEDEHEART registry
Source: PLoS One. 2021 Apr 8;16(4):e0249830. doi: 10.1371/journal.pone.0249830 (PMC8031406; doi:10.1371/journal.pone.0249830)
Supplement: S3 Table — A) Biomarkers with higher concentrations in men; B) Biomarkers with higher concentrations in women. (DOCX) [file pone.0249830.s006.docx]

**S3 Table. Results from the Lasso analysis. A) Biomarkers with higher concentrations in men; B) Biomarkers with higher concentrations in women.**

|  |  |  |  |  |
| --- | --- | --- | --- | --- |
| **A)** |  | **Odds ratio** | | |
|  |  |  |  |  |
|  |  |  |  |  |
| **Biomarker** | **Pathobiological importance** | **Crude** | **Model 1** | **Model 2** |
|  |  |  |  |  |
|  |  |  |  |  |
| MMP-3 | Atherogenesis | 3.53 | 4.39 | 4.72 |
| Complement C3 | Pro-inflammatory | 2.35 | 2.49 | 2.46 |
| Placenta growth factor | Atherogenesis | 2.27 | 3.59 | 3.93 |
| Galanin peptides | Glucose metabolism | 1.91 | 1.91 | 1.92 |
| Complement factor H | Anti-inflammatory | 1.65 | 1.93 | 2.04 |
| Myoglobin | Myocardial damage | 1.56 | 1.79 | 1.92 |
| Serine protease inhibitor | Pro-coagulatory | 1.34 | 1.35 | 1.34 |
| Apolipoprotein D | Lipid metabolism | 1.31 | 1.67 | 1.67 |
| PECAM 1 | Atherogensis | 1.30 | 1.19 | 1.24 |
| Carbonic anhydrase 1 | Myocardial function | 1.22 | 1.22 | 1.23 |
| Renin | RAAS-axis | 1.20 | 1.11 | 1.14 |
| IgA-1 chain C region | Pro-inflammatory | 1.13 | 1.20 | 1.21 |
| MCP 1 | Atherogenesis | 1.12 | 1.28 | 1.26 |
| ESM 1 | Angiogenesis | 1.12 | 1.11 | 1.18 |
| IgG-4 chain C region | Pro-inflammatory | 1.10 | 1.11 | 1.11 |
| IgA-2 chain C region | Pro-inflammatory | 1.09 | 1.14 | 1.14 |
| MB protein C | Acute phase reactant | 1.08 | 1.15 | 1.14 |
| MASP 2 | Pro-inflammatory | 1.05 | 1.09 | 1.08 |
| IgG-2 chain C region | Pro-inflammatory | 1.06 | - | - |
| TF pathway inhibitor | Anti-coagulatory | 1.02 | - | - |
| Pappalysin-1 | Atherogenesis | 1.01 | 1.02 | - |
| TNF-receptor SF 6 | Apoptosis | - | 1.16 | 1.16 |
| Beta-2-GP 1 | Anti-coagulatory | - | 1.12 | 1.18 |
| TPA | Anti-coagulatory | - | 1.08 | 1.09 |
| CC 3 | Pro-inflammatory | - | 1.08 | 1.09 |
| Alpha-1-antitrypsin | Acute phase reactant | - | 1.08 | 1.19 |
| Apolipoprotein B-100 | Lipid metabolism | - | 1.05 | 1.05 |
| MMP-12 | Atherogenesis | - | 1.04 | 1.05 |
| Vitamin K-dependent protein Z | Pro-coagulatory | - | 1.03 | 1.04 |
| Lipopolysaccharide-binding protein | Pro-inflammatory | - | - | 1.02 |
|  |  |  |  |  |

|  |  |  |  |  |
| --- | --- | --- | --- | --- |
| **B)** |  | **Odds ratio** | | |
|  |  |  |  |  |
|  |  |  |  |  |
| **Biomarker** | **Pathobiological importance** | **Crude** | **Model 1** | **Model 2** |
|  |  |  |  |  |
|  |  |  |  |  |
| Angiotensinogen | RAAS-axis | 0.34 | 0.25 | 0.24 |
| Ceruloplasmin | Acute phase reactant | 0.36 | 0.27 | 0.24 |
| Leptin | Adipokine | 0.39 | 0.34 | 0.33 |
| FABP 4 | Pro-inflammatory | 0.52 | 0.48 | 0.46 |
| Adiponectin | Adipokine | 0.54 | 0.55 | 0.53 |
| Galectin-3 | Myocardial function | 0.65 | 0.53 | 0.54 |
| Osteoprotegerin | Atherogenesis | 0.67 | 0.74 | 0.70 |
| FGF 23 | Hormone | 0.73 | 0.61 | 0.58 |
| Apolipoprotein C-I | Lipid metabolism | 0.75 | 0.57 | 0.56 |
| MMP-10 | Atherogenesis | 0.77 | 0.70 | 0.69 |
| Growth hormone | Hormone | 0.83 | 0.85 | 0.86 |
| CXCL 16 | Pro-inflammatory | 0.84 | 0.96 | - |
| SH binding globulin | Hormone | 0.85 | 0.86 | 0.84 |
| Prolactin | Angiogenesis | 0.89 | 0.77 | 0.77 |
| IL-27 subunit alpha | Proi-inflammatory | 0.93 | 0.75 | 0.74 |
| BNP | Myocardial function | 0.90 | 0.88 | 0.89 |
| MAMP | Atherogenesis | 0.95 | 0.92 | 0.89 |
| Agouti-related protein | Metabolism | - | 0.86 | 0.86 |
| Coagulation factor XIII B chain | Pro-coagulatory | - | 0.92 | 0.88 |
| Spondin-1 | Hormone | - | 0.96 | 0.88 |
| Fractalkine | Pro-inflammatory | - | 0.96 | 0.93 |
| DKK 1 | Hormone | - | 0.98 | 0.96 |
| CA 125 | Pro-inflammatory | - | 0.99 | 0.98 |
| VEGF D | Angiogenesis | - | 0.99 | 0.97 |
|  |  |  |  |  |

Model 1: adjusted for age, hypertension, diabetes, current smoking, renal failure, previous myocardial infarction, previous coronary revascularization, previous congestive heart failure, atrial fibrillation on the admission ECG, previous stroke, chronic obstructive pulmonary disease, dementia, previous or present cancer and peripheral vascular disease.

Model 2: additionally adjusted for ST-elevation myocardial infarction, pulmonary rales at admission and cardiogenic shock at admission.

OR >1 correspond to an increased probability of male sex. OR <1 correspond to an increased probability of female sex.

OR: odds ratio; MMP: Matrix metalloproteinase; PECAM 1: Platelet endothelial cell adhesion molecule 1; Ig: Immunoglobulin; MCP 1: Monocyte chemotactic protein 1; ESM-1: Endothelial cell-specific molecule-1; MB: Mannose-binding; MASP 2: Mannan-binding lectin serine protease 2; TF: Tissue factor; TNF-receptor SF 6: Tumor necrosis factor receptor superfamily member 6; GP: Glykoprotein; TPA: Tissue-type plasminogen activator; CC 3: C-C motif chemokine 3; FABP 4: Fatty acid-binding protein 4; FGF 23: Fibroblast growth factor 23; CXCL 16: C-X-C motif chemokine 16; SH: Sex hormone; IL: Interleukin; BNP: B-type natriuretic peptide; MAMP: Membrane-bound aminopeptidase P; DKK 1: Dickkopf-related protein 1; CA 125: Ovarian cancer-related tumor marker CA 125; VEGF D: Vascular endothelial growth factor D.
