# Supplementary material for: Sex-differences in circulating biomarkers during acute myocardial infarction: An analysis from the SWEDEHEART registry
Source: PLoS One. 2021 Apr 8;16(4):e0249830. doi: 10.1371/journal.pone.0249830 (PMC8031406; doi:10.1371/journal.pone.0249830)
Supplement: S4 Table — A) Biomarkers with higher concentrations in men; B) Biomarkers with higher concentrations in women. (DOCX) [file pone.0249830.s007.docx]

**S4 Table. Results from the Lasso analysis in subcohorts without and with previously known coronary artery disease. A) Biomarkers with higher concentrations in men; B) Biomarkers with higher concentrations in women.**

|  |  |  |  |
| --- | --- | --- | --- |
| **A)** |  | **Odds ratio** | |
|  |  |  |  |
|  |  |  |  |
| **Biomarker** | **Pathobiological importance** | **No CAD**  **(n=862)** | **CAD (n=237)** |
|  |  |  |  |
|  |  |  |  |
| MMP-3 | Atherogenesis | 5.20 | 2.77 |
| Galanin peptides | Glucose metabolism | 2.18 | 1.12 |
| Myoglobin | Myocardial damage | 1.95 | - |
| Apolipoprotein D | Lipid metabolism | - | 3.22 |
| IgA-2 chain C region | Pro-inflammatory | 1.10 | 1.08 |
| IgG-4 chain C region | Pro-inflammatory | 1.10 | 1.07 |
|  |  |  |  |
|  |  |  |  |
|  |  |  |  |
| **B)** |  | **Odds ratio** | |
|  |  |  |  |
|  |  |  |  |
| **Biomarker** | **Pathobiological importance** | **No CAD**  **(n=862)** | **CAD (n=237)** |
|  |  |  |  |
|  |  |  |  |
| Angiotensinogen | RAAS-axis | 0.30 | 0.27 |
| Ceruloplasmin | Acute phase reactant | 0.35 | 0.36 |
| Leptin | Adipokine | 0.27 | 0.65 |
| FABP 4 | Pro-inflammatory | 0.61 | 0.45 |
| Adiponectin | Adipokine | 0.54 | 0.53 |
| Galectin-3 | Myocardial function | 0.39 | 0.99 |
| Osteoprotegerin | Atherogenesis | 0.73 | - |
| FGF 23 | Hormone | 0.59 | 0.93 |
| Apolipoprotein C-I | Lipid metabolism | 0.66 | 0.48 |
| MMP-10 | Atherogenesis | 0.75 | 0.51 |
| Growth hormone | Hormone | 0.85 | - |
| SH binding globulin | Hormone | 0.82 | 0.91 |
| Prolactin | Angiogenesis | 0.73 | - |
| BNP | Myocardial function | 0.81 | - |
|  |  |  |  |

Only biomarkers with significant sex-differences in concentrations (Mann-Whitney test) are listed.

Adjustment was made for age, hypertension, diabetes, current smoking, renal failure, previous myocardial infarction, previous coronary revascularization, previous congestive heart failure, atrial fibrillation on the admission ECG, previous stroke, chronic obstructive pulmonary disease, previous or present cancer, peripheral vascular disease, ST-elevation myocardial infarction, pulmonary rales at admission and cardiogenic shock at admission (model 2).

OR >1 correspond to an increased probability of male sex. OR <1 correspond to an increased probability of female sex.

CAD: coronary artery disease; MMP: Matrix metalloproteinase; Ig: Immunoglobulin; RAAS: Renin-angiotensin-aldosterone; FABP 4: Fatty-acid binding protein 4; FGF 23: Fibroblast growth factor 23; SH: Sex hormone; BNP: B-type natriuretic peptide.
