# Supplementary material for: Sex-differences in circulating biomarkers during acute myocardial infarction: An analysis from the SWEDEHEART registry
Source: PLoS One. 2021 Apr 8;16(4):e0249830. doi: 10.1371/journal.pone.0249830 (PMC8031406; doi:10.1371/journal.pone.0249830)
Supplement: S6 Table — (DOCX) [file pone.0249830.s009.docx]

**S6 Table. Summary of selected studies investigating the associations of biomarker levels with sex, changes during myocardial infarction and sex-related differences in outcome.**

Publications had been selected considering the completeness of information relevant for the current analysis and do not represent the results of a systematic literature search. Up to two publications had been selected per population.

**Biomarkers with higher concentrations in men.**

|  |  |  |  |  |  |  |
| --- | --- | --- | --- | --- | --- | --- |
| **Biomarker** | **Reference** | **Population** | **n** | **Sex with higher concentrations** | **Change in MI** | **Difference in outcome** |
|  |  |  |  |  |  |  |
|  |  |  |  |  |  |  |
| MMP-3 | [1] | MI | 164 | M | ↓ | n.a. |
|  |  |  |  |  |  |  |
| Galanin peptides | [2] | Healthy | 79 | M | n.a. | n.a. |
|  |  |  |  |  |  |  |
| Myoglobin | [3] | Healthy | 7184 | M | n.a. | none |
|  |  |  |  |  |  |  |

For apolipoprotein D, immunoglobulin (Ig) A-2 chain C region and IgG-4 chain C region, no suitable studies could be identified.

M: Men; W: Women; MI: Myocardial infarction; n.a.: not assessed; MMP-3: Matrix metalloproteinase 3.

**Biomarkers with higher concentrations in women.**

|  |  |  |  |  |  |  |
| --- | --- | --- | --- | --- | --- | --- |
| **Biomarker** | **Author** | **Population** | **n** | **Sex with higher concentrations** | **Change in MI** | **Difference in outcome** |
|  |  |  |  |  |  |  |
|  |  |  |  |  |  |  |
| Angio- | [4] | Healthy | 445 | W | n.a. | n.a. |
| tensinogen | [5] | Healthy | 1557 | W | n.a. | n.a. |
|  |  |  |  |  |  |  |
| Cerulo- | [3] | Healthy | 7184 | W | n.a. | none |
| plasmin | [6] | Healthy | 9240 | W | n.a. | none |
|  | [7] | CAD | 3253 | W | ↑ | n.a. |
|  |  |  |  |  |  |  |
| Leptin | [3] | Healthy | 7184 | W | n.a. | Mortality ↓ (W) |
|  | [8] | Healthy | 1905 | W | n.a. | none |
|  | [9] | CAD | 981 | W | n.a. | none |
|  | [10] | CAD/MI | 1907 | W | n.a. | CV death/MI ↑ (W) |
|  | [11] | MI | 180 | W | n.a. | n.a. |
|  |  |  |  |  |  |  |
| FABP 4 | [12] | Healthy | 313 | W | n.a. | n.a. |
|  | [13] | CAD | 1069 | W | n.a. | none |
|  | [12] | CAD/MI | 507 | W | n.a. | n.a. |
|  |  |  |  |  |  |  |
| Adiponectin | [14] | Healthy | 1513 | W | n.a. | CV death/MI ↓ (M) |
|  | [15] | Healthy | 3439 | none | n.a. | n.a. |
|  | [16] | ACS | 3931 | W | n.a. | n.a. |
|  | [17] | MI | 156 | W | n.a. | Mortality ↑ (M) |
|  | [11] | MI | 180 | W | n.a. | Mortality ↑ (M) |
|  |  |  |  |  |  |  |
| Galectin-3 | [18] | Healthy | 7968 | W | n.a. | n.a. |
|  | [19] | Healthy | 1393 | W | n.a. | none |
|  | [20] | MI | 1342 | W | n.a. | n.a. |
|  |  |  |  |  |  |  |
| Osteo- | [3] | Healthy | 7184 | M | n.a. | none |
| protegerin | [15] | Healthy | 3439 | none | n.a. | n.a. |
|  | [21] | ACS | 897 | none | n.a. | n.a. |
|  |  |  |  |  |  |  |
| FGF 23 | [3] | Healthy | 7184 | W | n.a. | none |
|  | [22] | Healthy | 6547 | M | n.a. | none |
|  | [23] | MI | 88 | W | n.a. | n.a. |
|  |  |  |  |  |  |  |
| GH | [24] | Healthy | 4323 | W | none | Mortality ↑ (M) |
|  |  |  |  |  |  |  |
| SH-binding globulin | [25] | Healthy | 1384 | none | n.a. | n.a. |
|  |  |  |  |  |  |  |
| Prolaktin | [26] | Healthy | 3929 | W | n.a. | Mortality ↑ (M) |
|  |  |  |  |  |  |  |
| BNP | [27] | Healthy | 3697 | W | n.a. | n.a. |
|  | [3] | Healthy | 7184 | W | n.a. | none |
|  | [28] | ACS | 1565 | W | n.a. | n.a. |
|  | [29] | ACS | 3493 | W | ↑ | n.a. |
|  |  |  |  |  |  |  |

For matrix metalloproteinase-10 and apolipoprotein C-I, no suitable studies could be identified.

M: Men; W: Women; MI: Myocardial infarction; CAD: Stable coronary artery disease; ACS: Acute coronary syndrome; n.a.: not assessed; FABP 4: Fatty acid-binding protein 4; FGF 23: Fibroblast growth factor 23; GH: Growth hormone; SH: Sex hormone; BNP: B-type natriuretic peptide.

**References.**

# 1. Samnegård A, Silveira A, Tornvall P, Hamsten A, Ericsson CG, Eriksson P, et al. Lower serum concentration of matrix metalloproteinase-3 in the acute stage of myocardial infarction. J Intern Med. 2006;259: 530-536.

2. Fang P, Yu M, Gu X, Shi M, Zhu Y, Zhang Z, et al. Circulating galanin and galanin like peptide concentrations are correlated with increased triglyceride concentration in obese patients. Clin Chim Acta. 2016;461: 126-129.

# 3. Lau ES, Paniagua SM, Guseh JS, Bhambhani V, Zanni MV, Courchesne P, et al. Sex differences in circulating biomarkers of cardiovascular disease. J Am Coll Cardiol. 2019;74: 1543-1553.

4. Millen AME, Woodiwiss AJ, Gomes M, Michel F, Norton GR. Systemic angiotensinogen concentrations are independently associated with left ventricular diastolic function in a community sample. Am J Hypertens. 2018;31: 212-219.

5. Cooper R, Forrester T, Ogunbiyi O, Muffinda J. Angiotensinogen levels and obesity in four black populations. J Hypertens. 1998;16: 571-575.

6. Dadu RT, Dodge R, Nambi V, Virani SS, Hoogeveen RC, Smith NL, et al. Ceruloplasmin and heart failure in the Atherosclerosis Risk in Communities study. Circ Heart Fail. 2013;6: 936-943.

7. Grammer TB, Kleber ME, Silbernagel G, Pilz S, Scharnagl H, Lerchbaum E, et al. Copper, ceruloplasmin, and long-term cardiovascular and total mortality (the Ludwigshafen Risk and Cardiovascular Health Study). Free Radic Res. 2014;48: 706-715.

8. Martin SS, Blaha MJ, Muse ED, Qasim AN, Reilly MP, Blumenthal RS, et al. Leptin and incident cardiovascular disease: the Multi-ethnic Study of Atherosclerosis (MESA). Atherosclerosis. 2015;239: 67-72.

# 9. Ku IA, Farzaneh-Far R, Vittinghoff E, Zhang MH, Na B, Whooley MA. Association of low leptin with cardiovascular events and mortality in patients with stable coronary artery disease: the Heart and Soul Study. Atherosclerosis. 2011;217: 503-538.

# 10. Bickel C, Schnabel RB, Zeller T, Lackner KJ, Rupprecht HJ, Blankenberg S, et al. Predictors of leptin concentration and association with cardiovascular risk in patients with coronary artery disease: results from the AtheroGene study. Biomarkers. 2017;22: 210-218.

# 11. Ritsinger V, Brismar K, Malmberg K, Mellbin L, Näsman P, Rydén L, et al. Elevated levels of adipokines predict outcome after acute myocardial infarction: A long-term follow-up of the Glucose Tolerance in Patients with Acute Myocardial Infarction cohort. Diab Vasc Dis Res. 2017;14: 77-87.

# 12. Reiser H, Klingenberg R, Hof D, Cooksley-Decasper S, Fuchs N, Akhmedov A, et al. Circulating FABP4 is a prognostic biomarker in patients with acute coronary syndrome but not in asymptomatic individuals. Arterioscler Thromb Vasc Biol. 2015;35: 1872-1879.

13. von Eynatten M, Breitling LP, Roos M, Baumann M, Rothenbacher D, Brenner H. Circulating adipocyte fatty acid-binding protein levels and cardiovascular morbidity and mortality in patients with coronary heart disease: a 10-year prospective study. Arterioscler Thromb Vasc Biol. 2012;32: 2327-2335.

# 14. Laughlin GA, Barrett-Connor E, May S, Langenberg C. Association of adiponectin with coronary heart disease and mortality: the Rancho Bernardo study. Am J Epidemiol. 2007;165: 164-174.

# 15. Lew J, Sanghavi M, Ayers CR, McGuire DK, Omland T, Atzler D, et al. Sex-based differences in cardiometabolic biomarkers. Circulation. 2017;135: 544-555.

16. Wilson SR, Sabatine MS, Wiviott SD, Ray KK, De Lemos JA, Zhou S, et al. Assessment of adiponectin and the risk of recurrent cardiovascular events in patients presenting with an acute coronary syndrome: observations from the Pravastatin Or atorVastatin Evaluation and Infection Trial-Thrombolysis in Myocardial Infarction 22 (PROVE IT-TIMI 22). Am Heart J. 2011;161: 1147-1155.e1.

17. Kojima S, Funahashi T, Otsuka F, Maruyoshi H, Yamashita T, Kajiwara I, et al. Future adverse cardiac events can be predicted by persistently low plasma adiponectin concentrations in men and marked reductions of adiponectin in women after acute myocardial infarction. Atherosclerosis. 2007;194: 204-213.

18. de Boer RA, van Veldhuisen DJ, Gansevoort RT, Muller Kobold AC, van Gilst WH, Hillege HL, et al. The fibrosis marker galectin-3 and outcome in the general population. J Intern Med. 2012;272: 55-64.

19. Daniels LB, Clopton P, Laughlin GA, Maisel AS, Barrett-Connor E. Galectin-3 is independently associated with cardiovascular mortality in community-dwelling older adults without known cardiovascular disease: The Rancho Bernardo Study. Am Heart J. 2014;167: 674-682.e1.

20. Asleh R, Enriquez-Sarano M, Jaffe AS, Manemann SM, Weston SA, Jiang R, et al. Galectin-3 levels and outcomes after myocardial infarction: A population-based study. J Am Coll Cardiol. 2019;73: 2286-2295.

21. Omland T, Ueland T, Jansson AM, Persson A, Karlsson T, Smith C, et al. Circulating osteoprotegerin levels and long-term prognosis in patients with acute coronary syndromes. J Am Coll Cardiol. 2008;51: 627-633.

22. Kestenbaum B, Sachs MC, Hoofnagle AN, Siscovick DS, Ix JH, Robinson-Cohen C, et al. Fibroblast growth factor-23 and cardiovascular disease in the general population: the Multi-Ethnic Study of Atherosclerosis. Circ Heart Fail. 2014;7: 409-417.

23. Reindl M, Reinstadler SJ, Feistritzer HJ, Mueller L, Koch C, Mayr A, et al. Fibroblast growth factor 23 as novel biomarker for early risk stratification after ST-elevation myocardial infarction. Heart. 2017;103: 856-862.

24. Hallengren E, Almgren P, Engström G, Hedblad B, Persson M, Suhr J, et al. Fasting levels of high-sensitivity growth hormone predict cardiovascular morbidity and mortality: the Malmö Diet and Cancer study. J Am Coll Cardiol. 2014;64: 1452-1460.

# 25. Goodman-Gruen D, Barrett-Connor E. A prospective study of sex hormone-binding globulin and fatal cardiovascular disease in Rancho Bernardo men and women. J Clin Endocrinol Metab. 1996;81: 2999-3003.

26. Haring R, Friedrich N, Völzke H, Vasan RS, Felix SB, Dörr M, et al. Positive association of serum prolactin concentrations with all-cause and cardiovascular mortality. Eur Heart J. 2014;35: 1215-1221.

27. Kara K, Mahabadi AA, Geisel MH, Lehmann N, Kälsch H, Bauer M, et al. B-type natriuretic peptide: distribution in the general population and the association with major cardiovascular and coronary events--the Heinz Nixdorf Recall Study. Clin Res Cardiol. 2014;103: 125-132.

# 28. Wiviott SD, Cannon CP, Morrow DA, Murphy SA, Gibson CM, McCabe CH, et al. Differential expression of cardiac biomarkers by gender in patients with unstable angina/non-ST-elevation myocardial infarction: a TACTICS-TIMI 18 (Treat Angina with Aggrastat and determine Cost of Therapy with an Invasive or Conservative Strategy-Thrombolysis In Myocardial Infarction 18) substudy. Circulation. 2004;109: 580-586.

# 29. Morrow DA, de Lemos JA, Blazing MA, Sabatine MS, Murphy SA, Jarolim P, et al. Prognostic value of serial B-type natriuretic peptide testing during follow-up of patients with unstable coronary artery disease. JAMA. 2005;294:2866-2871.
