# Supplementary material for: Sex-differences in circulating biomarkers during acute myocardial infarction: An analysis from the SWEDEHEART registry
Source: PLoS One. 2021 Apr 8;16(4):e0249830. doi: 10.1371/journal.pone.0249830 (PMC8031406; doi:10.1371/journal.pone.0249830)
Supplement: S7 Table — (DOCX) [file pone.0249830.s010.docx]

**S7 Table. Cardiac troponin assays used during the study period.**

|  | |
| --- | --- |
| **Stockholm** | |
|  | |
|  | |
| cTnI (Beckman) | until September 2011 |
| cTnI (Dade Behring / Siemens) | until October 2012 |
| Hs-cTnT (Roche) | from October 2012 |
|  | |
|  | |
| **Lund** | |
|  | |
|  | |
| cTnT (Roche) | until December 2012 |
| Hs-cTnT (Roche) | from December 2012 |
|  | |
|  | |
| **Uppsala** | |
|  | |
|  | |
| cTnI (Abbott) | until June 2013 |
| Hs-cTnI (Abbott) | from June 2013 |
| cTnI (Dade Behring / Siemens) | entire study period |
|  | |

cTn: cardiac troponin; hs: high-sensitivity.
