# Supplementary material for: Sex-differences in circulating biomarkers during acute myocardial infarction: An analysis from the SWEDEHEART registry
Source: PLoS One. 2021 Apr 8;16(4):e0249830. doi: 10.1371/journal.pone.0249830 (PMC8031406; doi:10.1371/journal.pone.0249830)
Supplement: S1 Methods — (DOCX) [file pone.0249830.s011.docx]

**S Methods. Description of the Lasso analysis.**

**Introduction**

The Lasso analysis applied in the current investigation is an example of a penalized regression model. The idea is to basically fit a usual regression model, but reduce the freedom of each regression coefficient by ''shrinking'' it towards zero (or more precisely, punishing values far away from zero). In this way, a penalized predictor (i.e.biomarker) only ''counts as'' a fraction of an unpenalized one, making the classical 10:1 rule more within reach.

For the Lasso, the penalization is done by modifying the maximum likelihood procedure, instead maximizing the following function of the regression coefficients *ß* = (*ß*_1_, *ß*_2_,…, *ß*_p_), where *p* is the number of predictors:

log *L*_Lasso_ (*ß*) = log *L* (*ß*) - $\frac{1}{2}$*λ* $\sum_{j=1}^{p} ß_{j}$,

where L(*ß*) is the usual likelihood for the unpenalized regression model, and *λ* > 0 is a penalization parameter, chosen by the user. Thus, a large value of a regression coefficient will reduce the modified likelihood, making it a less attractive choice in the maximizing procedure.

A special feature of the Lasso is that some regression coefficients are typically shrunken all the way to zero. In this way, Lasso also performs automatic variable selection (biomarkers with regression coefficient zero are no longer in the model).

**Selection of λ**

The penalization parameter *λ* must be chosen somehow. Large values will shrink all coefficients to zero, whereas *λ* = 0 gives us back the classical regression model. Sensible choices are usually somewhere in-between.

We used repeated 10-fold cross-validation to select *λ*. This means that several possible values of *λ* were evaluated. For each such value, the data set was randomly split into 10 parts, so-called folds. For each fold, a lasso model was fitted by holding out this fold. Then, the model was evaluated on the held-out data by computing the c-statistic for censored data. By averaging these 10 c-statistics, one gets an estimate of true c-statistic for this value of *λ*. In the end, the *λ* with the largest c-statistic was chosen. As a by-product, one gets a realistic estimate of the true c-statistic, since the models have been evaluated on data not taking part in the model fitting.

The following slight modifications were applied to the above description of the selection of *λ*:

1. The whole cross-validation process was repeated 20 times, again averaging the results. This was done in order to minimize the effect of randomly splitting the data, making the results more repeatable.
2. Actually, the *λ* with the largest c-statistic was not chosen. Rather, we picked a slightly larger value (increasing the penalization somewhat), by taking the largest *λ* which gave a c-statistic that was no more than 1 standard error of the mean (of the estimated c-statistic) smaller than the best. The idea is that differences of that order could just as well be effects of chance (due to the random splitting), and so one could probably penalize a little extra without losing much.

**No shrinkage of clinical variables**

When clinical variables are added to a Lasso model, these are not shrunken (only the biomarkers are). This means that the models will contain all clinical variables together with a selection of biomarkers.

The idea behind this is to give the clinical variables greater priority. A biomarker can only enter the model if it adds predictive value above the clinical variables. Or, less formally expressed, in a ''competition'' between a clinical variable and a highly correlated biomarker, the clinical variable will always win.

**Reporting the results**

Since Lasso is really a usual regression model with a fancy model fitting process, the results can be summarized as for classical regression models, i.e. as odds ratios. However, many regression coefficients have been shrunken to zero. In the tables below, we only present results for the predictors that have ''survived'' the penalization (regression coefficient ≠ 0; odds ratio ≠ 1).

No standardization of the regression coefficients has been performed. In other words, each coefficient (or odds ratio) refers to a 1-unit change in the corresponding biomarker value, i.e. a doubling of the concentration.
